# Impact of Personal Protection Habits on the Spread of Pandemics: Insights from an Agent-Based Model

**DOI:** 10.1155/2021/6616654

**Published:** 2021-04-01

**Authors:** Lindsay Álvarez-Pomar, Sergio Rojas-Galeano

**Affiliations:** Universidad Distrital Francisco José de Caldas, Bogotá, Colombia

## Abstract

**Background:**

After several waves of spread of the COVID-19 pandemic, countries around the world are struggling to regain their economies by slowly lifting mobility restrictions and social distance measures applied during the crisis. Meanwhile, recent studies provide compelling evidence on how contact distancing, the use of face masks, and handwashing habits can reduce the risk of SARS-CoV-2 transmission. In this context, we investigated the effect that these personal protection habits can have in preventing new waves of contagion.

**Methods:**

We extended an agent-based COVID-19 epidemic model in a simulated community to incorporate the mechanisms of these aforementioned personal care habits and measure their incidence in person-to-person transmission. A full factorial experiment design was performed to illustrate the extent to which the interplay between these personal habits is effective in mitigating the spread of disease. A global sensitivity analysis was performed on the parameters that control these habits to further validate the results.

**Results:**

We found that observing physical distance is the dominant habit in reducing disease transmission, although adopting either or both of the other two habits is necessary to some extent to suppress a new outbreak entirely. When physical distance is not observed, adherence to the use of masks or handwashing has a significant decrease in infections and mortality, but the epidemic still unfolds. We also found that in all scenarios, the combined effect of adhering to the three habits is more powerful than adopting them separately.

**Conclusions:**

Our findings suggest that a broad adherence of the population to voluntary self-care habits would help contain unfold of new outbreaks. The purpose of our model is illustrative and contributes to ratify the importance of urging citizens to adopt the amalgam of personal care habits as a primary collective protection measure to prevent communities from returning to confinements, while immunisation is carried out in late stages of the pandemic.

## 1. Introduction

Countries around the world are facing the extraordinary challenge of recovering the economy after the crisis caused by the COVID-19 pandemic [1–3]. The crisis had a negative impact on the social, economic, and psychological conditions of the population due to the application of nonpharmaceutical interventions (NPI) aimed at restricting the mobility of people, thus reducing the risk of contagion by direct contact, e.g., total lockdown, home quarantine, isolation of confirmed cases, and closure of conglomerate facilities [4–7].

During the first year of the pandemic, the only measure to contain the spread of the disease was the periodic application of these NPIs; however, the disease has spread in most countries of the world with an oscillatory dynamics resembling a wave; the rise of a high peak of infections is followed by mandatory restrictions of mobility for the population, leading to a fall on the number of infections and on governments to lift restrictions, which in turn allows social interactions to increase, causing the infection curve to rise again. At the time of writing, many countries in Europe and America are struggling with second and third waves of contagion [8, 9] and even a fourth wave has affected some Asian countries, such as Hong Kong [10].

In this context, extending the duration of the intermediate periods of low contagion within each wave would be essential to achieve a continuous recovery of the economies and health services of the countries. The unprecedented rapid development of the COVID-19 vaccines [11], less than a year after the discovery of the SARS-CoV-2 genome sequence [12], raised hopes for rapid mass immunisation to slow down the transmission of the virus. However, implementing billions of doses vaccination has proven to be a great challenge [13] due to supply shortages [14], financial and political difficulties in allocation for poor countries [15], and the antivaccination attitude within the general population [16, 17], among other reasons.

Other areas of uncertainty persist, such as the efficacy of dose administration within vaccination implementation plans [14], duration of vaccine immunity [18], dynamics of antibody production against the new pathogen [19], and the potential for reinfection by new strains of the virus [20–22]. Consequently, the surge of new waves of contagion is likely to occur still in this late phase of the pandemic response, where person-to-person transmission, particularly in crowded meetings or in poorly ventilated spaces, would represent the main causes of risk [23–25].

Consequently, individual protection measures taken voluntarily by citizens, such as maintaining physical proximity distance, wearing facial protective equipment, and washing hands regularly, would be extremely important to minimise the risk of person-to-person contact, avoiding not only the spread of the disease but also the need to send communities back into confinement.

Now, compared to the socioeconomic cost inflicted by government-mandated NPIs, it can be argued that personal protection measures are apparently inexpensive (or at least easy to implement) and yet highly effective in preventing the spread of SARS-CoV-2 [26]. One study showed that washing hands 6–10 times a day reduces personal risk of infection by approximately 34% compared to people who wash hands less often [27]. Other studies have previously linked hand hygiene as an effective countermeasure against similar respiratory infectious diseases such as influenza [28, 29].

Similarly, since airborne transmission has been identified as the main route for the spread of SARS-CoV-2 [30], the habit of wearing a mask that was once ruled out as innocuous by the World Organization for Health [31] is now recommended as a mandatory personal habit that serves as a barrier to filter the enveloped droplet release mechanism of this respiratory virus [30, 32].

The beneficial filtering effect both inwards and outwards of masks made from different fabric materials has been documented [33]. Some studies found a reduced risk of infection for healthy mask users; but in addition, if infection occurs anyway, the masks reduce the amount of virus particles the susceptible person was exposed to, presumably leading to a mild or asymptomatic infection [34]. As a result, universal masking could be useful as a complementary mechanism to immunisation at the community level [35] while vaccination implementation plans develop.

Another observational study reported on the successful protection of a community by wearing face masks while attending a hair salon where two hairstylists (who also wore face protection) were subsequently diagnosed with COVID-19 and developed symptoms; none of these clients were infected with the disease [36]. A retrospective study of secondary infections in homes revealed that the use of masks by the index case and family contacts before the onset of the patient's symptoms produced a significant reduction in the risk of transmission [37].

Although there is still insufficient evidence to estimate the exact proportion of risk reduction provided by mask protection, a general trend of mitigating the epidemic has been noted in several countries after the majority of the community adopted this measure [38, 39]. Similar studies on the combination of measures involving physical contact distancing, a mask, and eye protection point to its effectiveness in preventing person-to-person transmission of SARS-CoV-2 [26]. It has been suggested that in community settings, masks appeared to be effective with and without hand hygiene or even more protective if both were taken together [40].

In light of this background, our study focuses on the following question. To what extent can personal protection habits be effective in containing the spread of COVID-19 contagion, in the absence of any other NPI or treatment, when examined within a controlled simulated environment? To address this question, we designed experiments on a simulation model of COVID-19 contagion dynamics. We note that several mathematical models have been developed for this purpose, mainly on the basis of the classic epidemiological compartment model SEIR (susceptible-exposed-infected-removed) of transmission of diseases from person to person [41]. Many of these models incorporate extended compartments designed for particular characteristics of this disease [4–6, 42–46], in an attempt to evaluate the effects of its epidemics.

For example, the SARIIqSq model of [44] defines compartments for quarantined and infected isolated susceptible individuals; the model was calibrated using data from the pandemic in India, and imposing social distance was found to be crucial in controlling the outbreak. Another mechanistic model, SAIUQR [45], proposes compartments for reported, unreported, and quarantined symptomatic patients, and after calibration with data from some regions of India, it found that quarantine also plays an important role for transmission mitigation. A related model, SAIU [46] with susceptible, asymptomatic, reported, and unreported symptomatic compartments, found that the reproduction number *R*_0_ can be controlled by reducing the rate of disease transmission. When this indicator falls to values below one (*R*_0_ < 1), the transmission decreases, which makes the epidemic finally disappear [47].

Other approaches use statistical time series models to forecast the number of positive COVID-19 cases. For example, the studies of [48, 49] concluded that these types of models (ARIMA, Holt's linear exponential smoothing methods, and autoregressive distributed delay) were useful to characterise the dynamics of the epidemic in eight major Western countries, finding that they are consisting with the predictions of traditional SEIR models.

An alternative approach to simulate the dynamics of contagion is agent-based models (ABM), which simulate microscopic rules of simultaneous spatial interactions between a population of agents allowing to replicate the complex dynamics of a wider variety of containment measures (e.g., [42, 43, 50–55]). We build upon an ABM simulation tool previously developed by our team to study the effects of several NPIs on a modified SIRE + CARDS epidemiological model [53]. In this study, we expand the model to account for the personal protection habits mentioned above. As our findings indicate, mitigation effects emerge indeed when the majority of the population adheres to these protection measures.

We note that the purpose of the resulting computational model is illustrative [56] and contributes to ratify the importance of reaching the entire community to spread the message about the collective benefits of the mass adoption of personal care against COVID-19, as a complement of NPIs and vaccination campaigns.

## 2. Methods and Tools

We extended the agent-based model of COVID-19 epidemics of [57], which proposes a SIRE + CARDS epidemic model considering four states: susceptible, infectious, recovered, and extinct. Infectious is actually viewed as a macrostate that includes confirmed, asymptomatic, risky, fatal, and serious conditions. The model simulates an artificial community of agents that represent people living within a simulated enclosure or territory, where contagion develops as a consequence of space-time interactions that occur during their daily routines. In addition, there are a number of optional NPIs available to enforce on the community, including lockdowns, quarantines, mass testing, and zonal isolation. For specific details of the agent-based design, epidemic model, transition events, and NPI dynamics, we refer the reader to [57].

The model was modified to account for individual voluntary protection measures related to personal health habits, that is, physical distance, the use of face masks, and regular handwashing. Consequently, each agent was designed with three different personal habit traits: social distancer (SD), mask user (MU), and hand washer (HW). The actual choice of traits for an arbitrary agent at the beginning of the simulation is controlled by three adjustable parameters that represent the willingness of the total population to adopt them, in a proportion between 0 and 100%.

Now, the incidence of these personal habits on the risk of contagion during person-to-person contact was modelled as follows. First, social distancing agents tend to divert their trajectory to avoid a close encounter with other surrounding agents. The actual occurrence of contact events would depend on the random spatial interactions that the agents have when they move around the environment during their daily activities, as the simulation unfolds.

Second, after considering the evidence suggesting the efficacy of face masks in preventing the transmission of SARS-CoV-2 [26, 30, 32, 33, 35–39], we define four possible contact configurations during a single contagion event involving a susceptible agent and an infectious agent, along with their associated risks of transmission: if neither agent wears a mask, the probability of contagion would be 90%; if the susceptible agent wears a mask but the infectious agent does not, the probability of infection would be 50%; if the infectious agent wears a mask but the susceptible agent does not, the probability of contagion would be 30%; and the last case corresponds to both agents wearing masks, with a probability of contagion of 10%.

Third, and again taking into account recent studies suggesting the benefits of handwashing in the prevention of diseases caused by coronaviruses [27, 28], we define an additional reduction in the risk of contagion by a 30% factor if the susceptible agent involved in any of the contagion events described above happens to be a hand washer (e.g., in the case where the infectious agent wears masks but the susceptible does not, the risk decreases from 30% to 9%).

Given that the purpose of our study is to evaluate the effect that these habits may have in mitigating the epidemic, we decided to suspend the application of any of the other NPIs available in the model and experiment with simulations where the population adheres to the habits of SD, MU, and HW to some extent. For each of these traits, we define willingness parameters modelling scenarios where no one observes the habit (0%), approximately half of the population adopts it (50%), or everyone adheres to it (100%). The different permutations of the tuple of proportions (SD%, MU%, and HW%) produce twenty-seven scenarios starting with the organic “do nothing” scenario (0%, 0%, 0%) up to the ideal “everybody adheres to” scenario (100%, 100%, 100%) plus the in between permutations (0%, 0%, 50%), (0%, 0%, 100%), and (0%, 50%, 0%).

Besides, our analysis will consider the following epidemic indicators:

1. Mortality. This measure is the cumulative count of deaths in the population. Notice that in our model, all deaths are due to COVID-19, and no births or immigrations are taken into account during the simulation timeline. Thus, this indicator determines the mortality rate.
2. Cases. This measure is the cumulative count of infections in the population. Since the population evolves within a controlled environment where both symptomatic and asymptomatic patients can be traced, this would be in fact the actual number of cases due to the disease. This indicator is associated to the infection fatality rate (IFR).
3. Confirmed cases. This measure is the cumulative count of confirmed infection cases. Given that in our simulations, we do not allow the application of massive test interventions, and these cases correspond to symptomatic patients whose disease worsens to severe or fatal states that require hospital care, where upon admission, are reported as diagnosed. This indicator is associated to the case fatality rate (CFR) which is usually an overestimated representative of incidence rate (i.e., CFR and IFR).
4. Recovered. This measure is the cumulative count of agents that have recovered from illness. The model assumes that upon recovery, immunity to disease is acquired and reinfection will not occur. Therefore, this indicator is related to the survival rate and the herd immunity rate.

The derived model was developed in the NetLogo programming language version 6.1.0; the source-code and user documentation have been released openly at http://modelingcommons.org/browse/one_model/6423. There, the model can be run online or downloaded for local execution.

## 3. Simulation Results

The settings for the experiments, including properties for the simulated community, disease, NPIs, and running parameters, are given in Table 1 (note that for the sake of completeness, we included settings for authority-enforced NPIs, although they were disabled during the simulations). A single simulation begins at 00 hours on day 0 and lasts until 00 hours on day 60. At 12 hours on day 0, an outbreak is simulated that causes the infection of 5% of the population. From that moment on, the simulation unfolds according to the rules designed for the model and the random local interactions that occur during the movement of the agents.

### 3.1. Effect in “Flattening the Curve”

**Figure 1:**
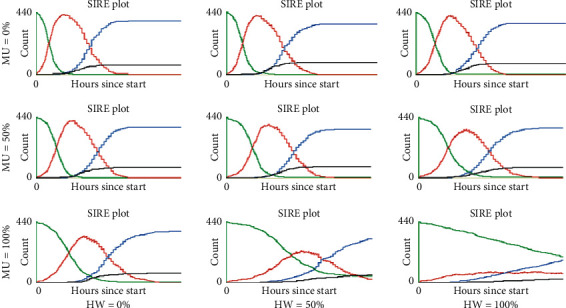
Representative SIRE curves for scenarios with SD = 0% and different combinations of MU and HW proportions (green, susceptible; red, infectious; blue, recovered; black, extinct).

**Figure 2:**
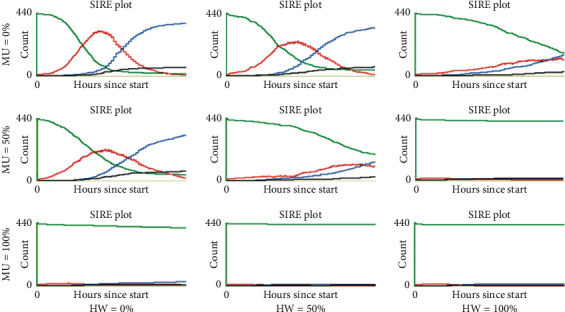
Representative SIRE curves for scenarios with SD = 50% and different combinations of MU and HW proportions (green, susceptible; red, infectious; blue, recovered; black, extinct).

**Figure 3:**
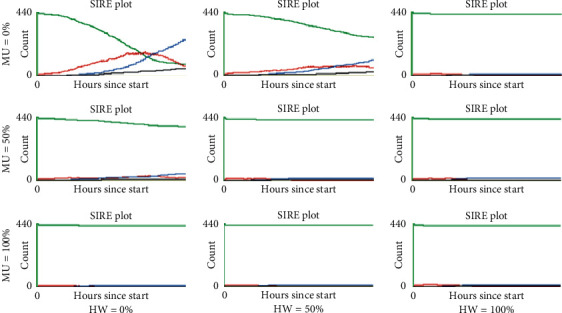
Representative SIRE curves for scenarios with SD = 100% and different combinations of MU and HW proportions (green, susceptible; red, infectious; blue, recovered; black, extinct).

Figures 1–3 show an illustration of the SIRE curves obtained in a single representative execution for each of the 27 scenarios. First of all, Figure 1 shows that the majority of the curves when no physical distancing is adopted (SD = 0%) exhibit the typical development of an uncontrolled epidemic, that is, an exponential growth of the infectious curve (red) reaching an early peak in the first third of the simulation. Only in scenarios where all agents wear mask protection (MU = 100%) and half or more of the agent population wash their hands regularly (HW = 50% or 100%), the infectious curve developed a flattened shape that indicates a mitigation on the speed of contagion.

On the other hand, Figure 2 shows that a 50% increase in agents who adhere to social distance (SD = 50%) has a notable effect on mitigating the infectious curve. Even when no other habit is adopted (MU = 0% and HW = 0%), the peak is reduced and moved towards the middle of the simulation timeline. When some other habit is adopted (MU = 50% or HW = 50%), the curve flattens more drastically. Furthermore, when more than 50% of the population adopts the use of masks or washes their hands or both (MU = 100% or HW = 100%), the transmission of the infection is suppressed, even from the early stages of the simulation.

Last, Figure 3 shows that all scenarios where everybody adheres to maintain physical distance (SD = 100%) and abide by the other habits to some extent (MU *>* 0% and HW *>* 0%) produce the suppression of contagion. Here, even when no one wears masks or washes their hands (MU = 0% and HW = 0%), the infectious curve clearly flattens out and shifts towards the end of the simulation.

### 3.2. Effect on the Epidemic Indicators

For each of the 27 scenarios, we performed 30 repetitions. Then, at the end of the simulation run (day 60), we collected average statistics from the aforementioned epidemic indicators. We focus the analysis of these results on evaluating the effect of variations in the estimated ratios of the population that adopts personal care habits, that is, we compare the results of the epidemic indicators for a series of (SD, MU, and HW) variations. To do this, we define three scenarios as a baseline: in the first, no one adopts any habit (0%, 0%, 0%); in the second, half of the population complies with the physical distance but does not wear a mask or wash their hands (50%, 0%, 0%); finally, the third baseline scenario assumes that everyone observes the physical distance, but again, no one wears masks or washes their hands (100%, 0%, 0%). The differences in behaviour between the reference scenarios and the variations were evaluated using the Mann–Whitney *U* statistical test (Supplementary Materials).

Next, we present plots with the results of the epidemic indicators of interest. Some periodic patterns are seen when examining groups of three boxes from left to right (a box with a higher average, followed by a box in the middle and a box with a lower average); such patterns are simply an artifact of the order of presentation of the parameters in the permutation tuple (SD, MU, and HW); we observe that when plotting the permutations in different orders, similar ladder patterns are obtained (with cycles of every three scenarios).

### 3.3. Mortality

**Figure 4:**
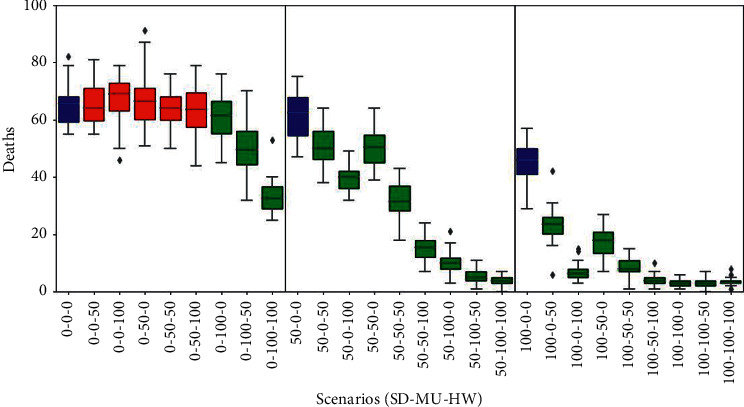
Mortality results. Box plots represent average and standard deviation of the cumulative count of deaths in each scenario. Scenarios are labelled according to the willingness of agents to adhere to personal health habits such as social distance, mask user, or hands washer (SD%, MU%, and HW%). Baseline scenarios were defined varying the proportion of social distancers in the population assuming no agents adopt using masks or washing hands. In each panel, the baseline scenario is coloured blue (left, 0%-0%-0%; middle, 50%-0%-0%; right, 100%-0%-0%). Scenarios with statistical significant difference compared to their corresponding panel baseline (blue) are coloured green (*p* value *<*0*:*05); otherwise, they are coloured red.

The behaviour of this epidemic indicator in all simulated scenarios is shown in Figure 4. The left panel shows scenarios where no one observes physical distancing (SD = 0%). No significant differences were found in the first 5 scenarios with respect to the (0%, 0%, 0%) baseline (number of deaths around 65, that is, 16% of the population). Only the last three scenarios in that panel, where everyone wears face masks (MU = 100%), showed a significant decrease in the number of deaths, with the steepest drop to almost a half when everyone also washed their hands (HW = 100%).

The middle panel shows scenarios where half the population adheres to physical distancing (SD = 50%). Within each group of 3 cells, a trend of deaths decreasing as the percentage of HW increases can be seen. In any case, the number of deaths in all scenarios drops significantly compared to the reference scenario (50%, 0%, 0%) for this panel. As expected, the last group of three scenarios obtained the lowest values, nearly less than 10 deaths (that is, 2.5% of the population).

The right panel shows scenarios where the entire population observes physical distancing (SD = 100%). A similar pattern can be observed to the results of the central panel, since compared to the baseline scenario (100%, 0%, 0%); all other scenarios exhibited a significant reduction in mortality, including the last six with death ratios lower than 2.5% of the population. These results are corroborated by the black extinct curve of the SIRE graphs of Figure 3, which is seen rising very low or even not rising at all.

### 3.4. Cases

**Figure 5:**
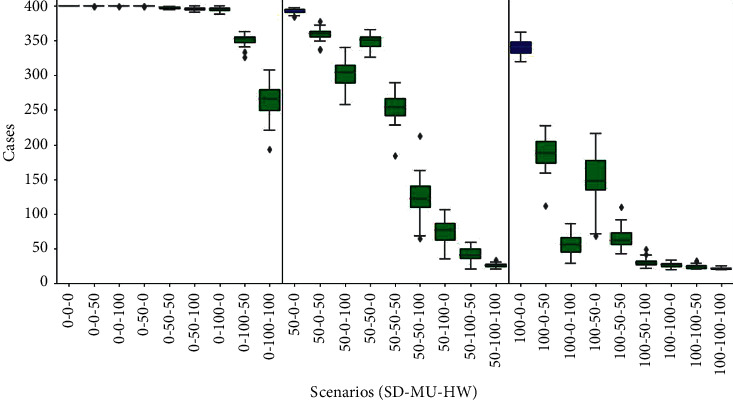
Cases results. Box plots represent average and standard deviation of the cumulative count of infections in the population. Scenarios are labelled according to the willingness of agents to adhere to personal health habits such as social distance, mask user, or hands washer (SD%, MU%, and HW%). Baseline scenarios were defined varying the proportion of social distancers in the population assuming no agents adopt using masks or washing hands. In each panel, the baseline scenario is coloured blue (left, 0%-0%-0%; middle, 50%-0%-0%; right, 100%-0%-0%). Scenarios with statistical significant difference compared to their corresponding panel baseline (blue) are coloured green (*p* value *<*0*:*05); otherwise, they are coloured red.

The behaviour of this epidemic indicator in all simulated scenarios is shown in Figure 5. The three panels are organised as before: SD = 0%, scenarios are plotted in the left panel, SD = 50%, scenarios in the middle panel, and SD = 100%, scenarios in the right panel. Regarding the left panel, a significant drop in the number of cases is clearly seen in the scenarios (0%, 100%, 50%) and (0%, 100%, 100%), in about 350 and 270 cases, respectively; the difference is not noticeable with respect to the other scenarios where the number of cases is close to the entire population (about 400 agents). The central panel displays a significant decrease in the number of cases for all scenarios. The largest drop is observed between the scenario (50%, 50%, 50%) and the scenario (50%, 50%, 100%), to around half of the cases (from 250 to 120).

Last, the right panel shows a pattern similar to the middle panel, although more notable; compared to the baseline (100%, 0%, 0%), for this panel, the other scenarios exhibited a significant reduction in cases, approaching 25 cases in the last four scenarios (6.25% of the population). These low rates are explained by the fact that, as shown by Figures 2 and 3, the red infectious curve in some of these scenarios flattens out, and, therefore, the contagion is in the stage of initial growth when the indicator was collected at the end of the simulated timeline (day 60); moreover, in some other cases, the curve was completely suppressed.

### 3.5. Confirmed Cases

**Figure 6:**
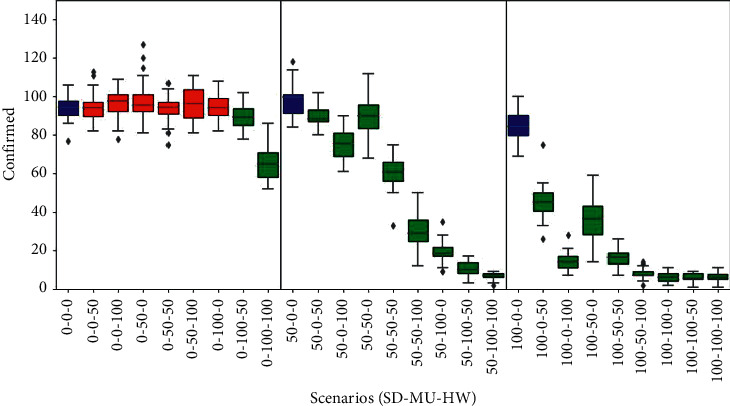
Confirmed cases results. Box plots represent average and standard deviation of the cumulative count of only those cases reported as diagnosed. Scenarios are labelled according to the willingness of agents to adhere to personal health habits such as social distance, mask user, or hands washer (SD%, MU%, and HW%). Baseline scenarios were defined varying the proportion of social distancers in the population assuming no agents adopt using masks or washing hands. In each panel, the baseline scenario is coloured blue (left, 0%-0%-0%; middle, 50%-0%-0%; right, 100%-0%-0%). Scenarios with statistical significant difference compared to their corresponding panel baseline (blue) are coloured green (*p* value *<*0*:*05); otherwise, they are coloured red.

The behaviour of this epidemic indicator in all simulated scenarios is shown in Figure 6. A similar pattern with the mortality plots of Figure 4 can be seen in all three panels. In contrast to mortality, the value of confirmed cases is higher than deaths (see the three baseline scenarios that come close to around 90 confirmed cases on average). The results of confirmed cases are much lower compared to the results of real cases because in these simulations, we do not apply massive tests, voluntary isolation, or home quarantine; therefore, the diagnosed cases correspond only to those admitted (and reported as cases) in the hospital due to complications with the disease.

This dynamics matches the CFR overestimation of the IFR, mentioned before. For instance, comparing the (0%, 0%, 0%) scenarios in Figure 5 vs. Figure 6, we got IFR = 17% (68/400) vs. CFR = 72% (68/95); another example occurs in (100%, 0%, 0%) where IFR = 12% (47/400) vs. CFR = 55% (47/85).

### 3.6. Recovered

**Figure 7:**
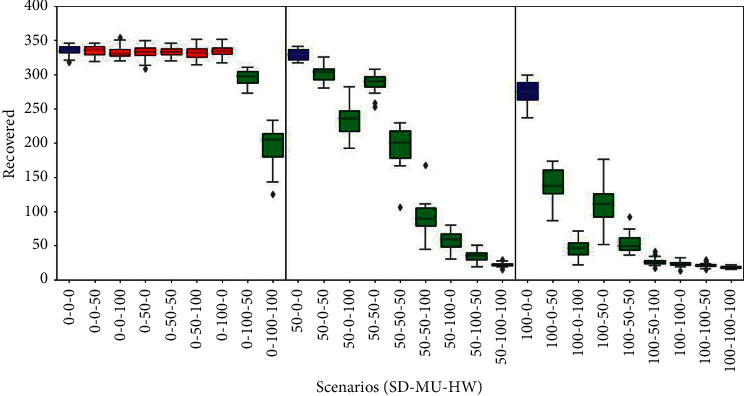
Recovered results. Box plots represent average and standard deviation of the cumulative count of agents that recovered from disease. Scenarios are labelled according to the willingness of agents to adhere to personal health habits such as social distance, mask user, or hands washer (SD%, MU%, and HW%). Baseline scenarios were defined varying the proportion of social distancers in the population assuming no agents adopt using masks or washing hands. In each panel, the baseline scenario is coloured blue (left, 0%-0%-0%; middle, 50%-0%-0%; right, 100%-0%-0%). Scenarios with statistical significant difference compared to their corresponding panel baseline (blue) are coloured green (*p* value *<*0*:*05); otherwise, they are coloured red.

The behaviour of this epidemic indicator in all simulated scenarios is shown in Figure 7. Here again, similar patterns appeared. In the left panel (SD = 0%), only the scenarios (0%, 100%, 50%) and (0%, 100%, 100%) showed a significant decrease in the recovered agents compared to the baseline scenario, dropping from around 340 to 300 and 200, respectively. Furthermore, in the central (SD = 50%) and right (SD = 100%) panels, significant reductions are observed in all scenarios compared to the baselines, with even more pronounced falls in the scenarios where half or more of the population adopt either or both of the other two measures (MU and HW), reaching values of up to approximately only 20 agents (that is, 5% of the population). The decrease in the recovered agents arises as a consequence of the mitigating or suppressing effects of these personal protection habits, which implies that fewer infections occurred, as observed in the SIRE curves shown in Figures 2 and 3.

## 4. Sensitivity Analysis

A global sensitivity analysis was conducted to identify how the uncertainty about the model parameters associated with personal protection habits, that is, the percentage of agents willing to comply with physical distance (SD), the use of a mask (MU), and handwashing (HW), influences the variability of the outcome of the epidemic indicators collected at the end of a simulation, that is, the number of deaths, cases, confirmed cases, and recovered cases. For this purpose, we used the Sobol–Saltelli method [58, 59] to quantify measures of main (individual) and total (combined) order effects. The range of variation of the parameters was established between 0% and 100%, and the number of initial trials of the Sobol sequence was established at 200, which for 3 variables produce a total of 1000 samples of combinations uniformly distributed in the parameter space. The sensitivity indices were calculated using Python software libraries [60, 61].

**Figure 8:**
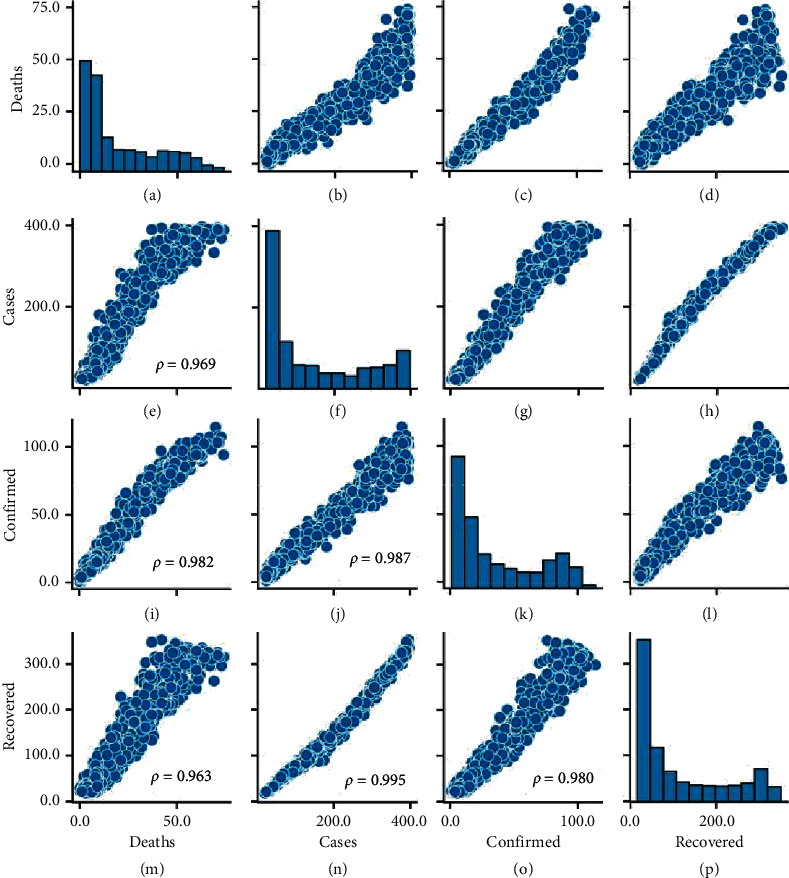
Pairwise correlations and frequency distribution of output variables, reported with *p* < 0.001.

We run simulations for each of the 1000 parameter combinations. The results of the outcome variables in these simulations are shown in the pairwise scatter plots of Figure 8. Positive correlations emerge between cases, confirmed cases, and deaths, as expected; an increase in the number of infections produces a higher number of hospitalisations and, consequently, a higher mortality rate; likewise, more patients would eventually recover. Furthermore, the correlation between deaths and recovered can be explained conditioned on the growth of cases; more deaths occur when there are more infections, which in turn implies that more agents will eventually recover.

On the other hand, the frequency histograms on the diagonal of the grid show skewed distributions of the results towards the lowest values, which suggests that most combinations of parameters produce a total effect that favours the mitigation of epidemics, in terms of infected agents. Another relevant observation is that only for the variable cases, several simulations reached an extreme contagion scenario where the entire population was infected, represented by the bar farthest to the right of the histogram (the third highest, in fact).

**Figure 9:**
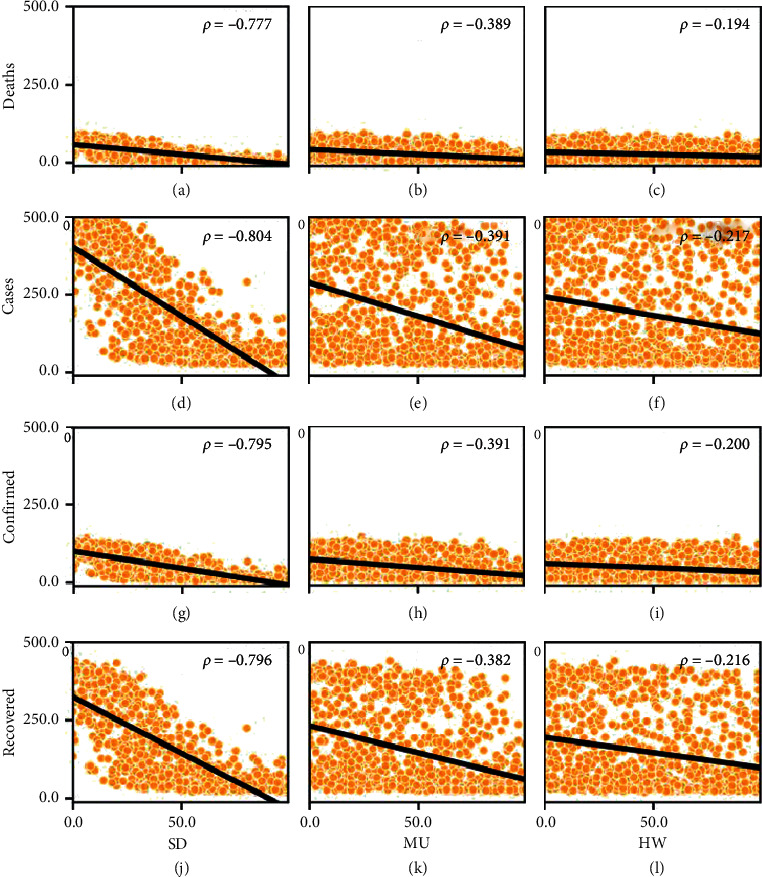
Correlation analysis between outcome variables and input parameters, reported with *p* < 0.001.

We also examined the possible linear relationships between the input parameters and the result variables in the 1000 simulations; these are shown in the scatter plots of Figure 9. All the pairwise associations between each personal habit parameter (SD, MU, and HW) and each epidemic indicator obtained a negative linear correlation. However, the strongest correlations are found between SD and each outcome variable, highlighting the dominant role physical distancing plays in reducing disease transmission.

The correlation between MU and the outcome variables is weak (almost half that of SD), while HW shows an even weaker association (almost a quarter of SD), suggesting that the mass adoption of mask use alone or handwashing alone would have a positive effect in reducing contagion but would probably be insufficient to stop the unfold of the epidemics. As a side note, the negative correlation with the variable recovered could be due to the decrease in infected agents that will eventually recover, rather than these habits having some effects on the deterioration in the progression of the disease. On the contrary, the negative correlation with the rest of the variables is explained because these habits help reduce the number of transmissions between agents.

**Figure 10:**
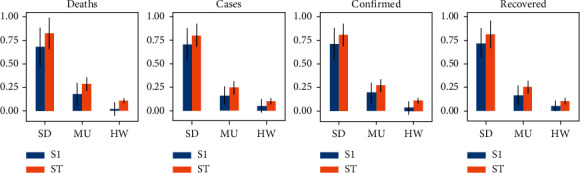
Sensitivity indices for first (S1) and total order (ST) variabilities of input parameters on each of the outcome variables, with a 95% confidence interval.

Last, we report the Sobol sensitivity indices for the SD, MU, and HW parameters as shown in Figure 10. The results corroborate some of the aforementioned observations. It is evident that physical distance (SD) is the most critical factor affecting the variance of each outcome variable, whether it is considered alone (first order effect, S1 = 0.7) or even more dramatic when interacting with two other habits (total order effect, ST = 0.8). Regarding the use of a mask (MU), it has a notable but small first order effect, which is more advantageous when interacting with the other two habits. Regarding handwashing, its first order effect on all variables is negligible; however, when interacting with the other two habits, the effect on the outcome variables becomes significant (parameters with sensitivity indices greater than 0.05 are considered significant [62]).

These findings imply that the habits of wearing masks and washing hands must be strongly linked to the willingness to maintain social distance; otherwise, its effect in helping to control epidemics would be marginal. They also indicate that persuading the community to adopt all three personal protection habits simultaneously is in fact a more powerful measure to reduce the impact of epidemics than developing adherence to just one of them.

## 5. Concluding Remarks

We have illustrated the interplay between maintaining physical distance, wearing face masks, and regular handwashing, with an agent-based model of the spread of the epidemic in an artificial community. Our results indicate that the almost universal adoption of one of these three habits alone would not be enough to mitigate the surge of new waves of contagion. Maintaining physical distance between agents plays a dominant role in reducing risk. However, a more powerful impact can be achieved if the community simultaneously adheres to the other two habits to some extent. These findings are in line with other ABM studies that highlight the positive effect of combining these measures in lieu of restricting population mobility to mitigate the pandemic [50, 63–65].

Reaching out to the community to persuade them to 100% adopt these habits can be difficult due to many factors related to personal beliefs, socioeconomic conditions, and collective idiosyncrasies. Therefore, public health education campaigns would play a key role in reinforcing collective awareness of the risks of virus transmission in the late phase of the response to the pandemic, when new waves of contagion are likely to emerge. Emphasising how these measures can help prevent these risks to avoid returning to lockdowns [66] would be important, even during the current implementation of vaccination plans, since in many countries, herd immunity may take longer to achieve due to delays in the acquisition or administration of the vaccine or due to the uncertainties about the period necessary to develop cellular immunity and the duration of antibodies to protect against reinfection after inoculation.

Therefore, it is important that authorities clearly communicate the evidence, uncertainties, risks, and particularities of these personal protection strategies, without creating the feeling of false dilemmas [67] while aiming to persuade the public for a greater adoption. Along these lines, the role of the media in communicating the prevalence of the coronavirus has previously been related to an impact on the decline of the disease [68].

Finally, extending the agent-based model to take into account additional realistic characteristics of the SARS-CoV-2 contagion, including infections through contaminated surfaces, population structure, spatial stratification, contact networks, antibodies generation, and other sources of heterogeneity between individuals, pose interesting challenges from a computational perspective [69]. Furthermore, from a modelling point of view, the question of habit formation constitutes a compelling line of research, as it ultimately depends on how people react and adhere to them, which might vary between segments of the population; hence, designing mechanisms allowing the emergence of willingness, as a consequence of changes in individual beliefs, media exposing, and fluctuations in the popular consciousness, is also appealing.

## Figures and Tables

**Table 1:** Settings used in the simulation tool to perform the experiments.

Parameter	Description	Value
City and population settings		
Population size	Total number of simulated people (agents)	400
Zones	Number of residential zones	9
Days	Period of observation days (simulation length)	60 days
% high risk	% of population with comorbidities	30%
Hospital beds	Total number of hospital beds available	12
ICU beds	Total number of ICU beds available	2
Ambulances zone	Number of ambulances (sentinels) per zone	1

Disease settings		
Average duration	Average day period to recover from illness	18 days
% asymptomatic	% of patients showing no or mild symptoms	50%

NPI settings (authority enforced, not used)		
Total lockdown	Enforce stay-at-home order for the entire population	Off
% permits	% of mobility permits when lockdown is activated	Not used
Case isolation	Confirmed cases are isolated at home	Off
Home quarantine	Housemates of confirmed cases are also isolated	Off
Zone enforcing	Restraint mobility of agents within zones only	Off
Pick zone	Zone id number	Not used
Sentinel testing	Enable mass testing by ambulance sentinels	Off
Zonal	Restraint mobility of sentinels within zones	Off

NPI settings (personal habits)		
Social distancing	Maintain a minimum physical distance with others (SD)	On
% willing	Estimated % of population willing to comply with SD	(0, 50, 100)
% mask users	Estimated % of population willing to use masks (MU)	(0, 50, 100)
% hand washers	Estimated % of population willing to wash hands (HW)	(0, 50, 100)

## Data Availability

The model used in this study was developed in the NetLogo programming language version 6.1.0; the source-code has been released openly and can be run online or downloaded for local execution at http://modelingcommons.org/browse/one_model/6423. A user guide of the simulation tool is also openly available at http://modelingcommons.org/file/download/6423?file_id=3772.

## Supplementary Materials

Supplementary Materials Mortality hypothesis tests for differences. Heatmaps represent counts and *p* values obtained by the Mann–Whitney *U* statistical test for permutations (SD%, MU%, and HW%) with varying proportions of social physical distance, mask using, and handwashing adoption by the population. Blue, baseline scenario; red, accept; green, reject. Cases hypothesis tests for differences. Heatmaps represent counts and *p* values obtained by the Mann–Whitney *U* statistical test for permutations (SD%, MU%, and HW%) with varying proportions of social physical distance, mask using, and handwashing adoption by the population. Blue, baseline scenario; red, accept; green, reject. Confirmed cases hypothesis tests for differences. Heatmaps represent counts and *p* values obtained by the Mann–Whitney *U* statistical test for permutations (SD%, MU%, and HW%) with varying proportions of social physical distance, mask using, and handwashing adoption by the population. Blue, baseline scenario; red, accept; green: reject. Recovered hypothesis tests for differences. Heatmaps represent counts and *p* values obtained by the Mann–Whitney *U* statistical test for permutations (SD%, MU%, and HW%) with varying proportions of social physical distance, mask using, and handwashing adoption by the population. Blue, baseline scenario; red, accept; green, reject.
