# Supplementary material for: Impact of Personal Protection Habits on the Spread of Pandemics: Insights from an Agent-Based Model
Source: ScientificWorldJournal. 2021 Apr 1;2021:6616654. doi: 10.1155/2021/6616654 (PMC8028727; doi:10.1155/2021/6616654)

**Supplementary material**

1. **Mortality hypothesis tests for differences.** Heatmaps represent counts and *p*-values obtained by the Mann-Whitney U statistical test for permutations (SD%, MU%, HW%) with vary-ing proportions of social physical distance, mask using and hand washing adoption by the population. Blue: baseline scenario, red: accept, green: reject.


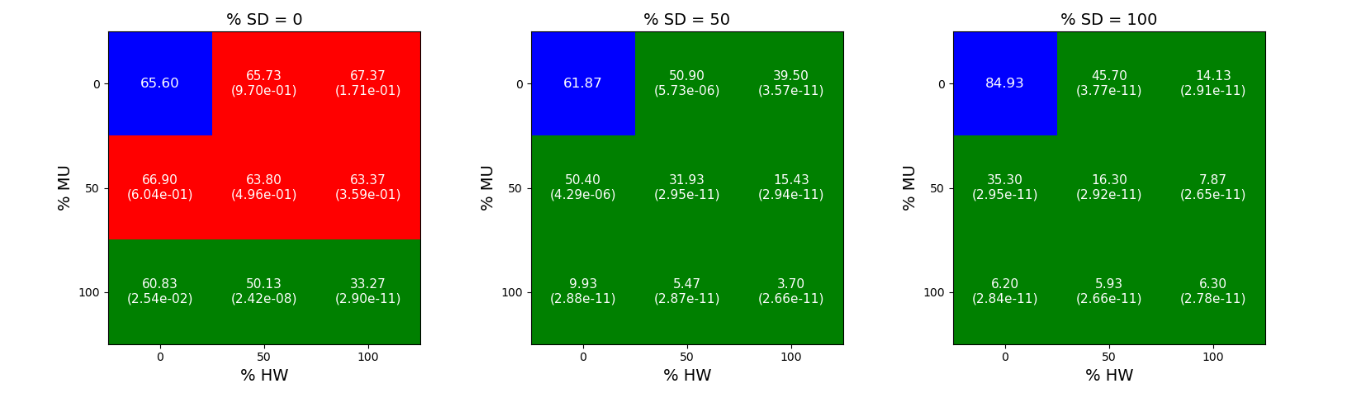


1. **Cases hypothesis tests for differences.** Heatmaps represent counts and *p*-values obtained by the Mann-Whitney U statistical test for permutations (SD%, MU%, HW%) with varying pro-portions of social physical distance, mask using and hand washing adoption by the population. Blue: baseline scenario, red: accept, green: reject.


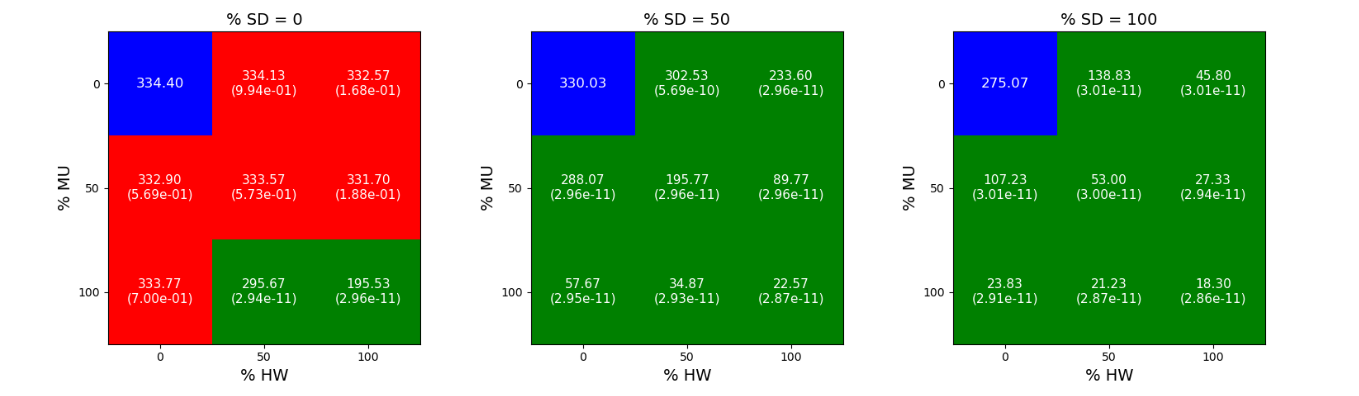


18 of 19

1. **Confirmed cases hypothesis tests for differences.** Heatmaps represent counts and *p*-values obtained by the Mann-Whitney U statistical test for permutations (SD%, MU%, HW%) with varying proportions of social physical distance, mask using and hand washing adoption by the population. Blue: baseline scenario, red: accept, green: reject.


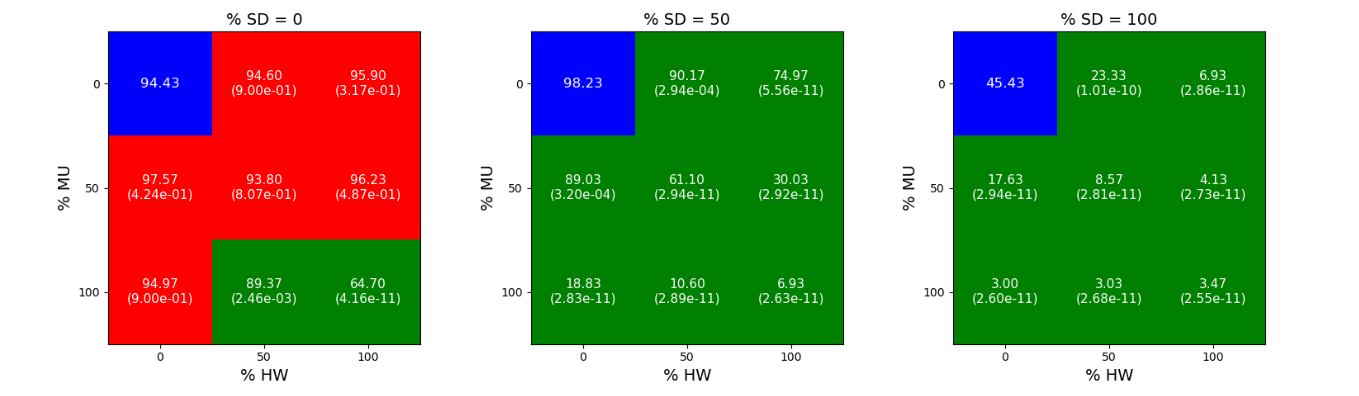


1. **Recovered hypothesis tests for differences.** Heatmaps represent counts and *p*-values obtained by the Mann-Whitney U statistical test for permutations (SD%, MU%, HW%) with vary-ing proportions of social physical distance, mask using and hand washing adoption by the population. Blue: baseline scenario, red: accept, green: reject.


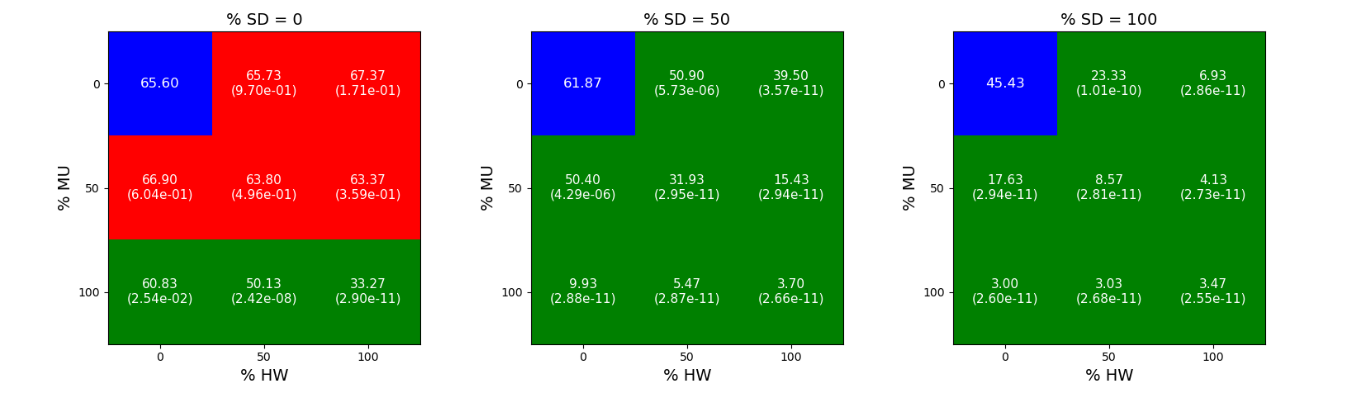

Supplement: Supplementary Materials — Mortality hypothesis tests for differences. Heatmaps represent counts and p values obtained by the Mann–Whitney U statistical test for permutations (SD%, MU%, and HW%) with varying proportions of social physical distance, mask using, and handwashing adoption by the population. Blue, baseline scenario; red, accept; green, reject. Cases hypothesis tests for differences. Heatmaps represent counts and p values obtained by the Mann–Whitney U statistical test for permutations (SD%, MU%, and HW%) with varying proportions of social physical distance, mask using, and handwashing adoption by the population. Blue, baseline scenario; red, accept; green, reject. Confirmed cases hypothesis tests for differences. Heatmaps represent counts and p values obtained by the Mann–Whitney U statistical test for permutations (SD%, MU%, and HW%) with varying proportions of social physical distance, mask using, and handwashing adoption by the population. Blue, baseline scenario; red, accept; green: reject. Recovered hypothesis tests for differences. Heatmaps represent counts and p values obtained by the Mann–Whitney U statistical test for permutations (SD%, MU%, and HW%) with varying proportions of social physical distance, mask using, and handwashing adoption by the population. Blue, baseline scenario; red, accept; green, reject. [file 6616654.f1.docx]
